# Overstimulation of the ergoreflex—A possible mechanism to explain symptoms in long COVID

**DOI:** 10.3389/fcvm.2022.940832

**Published:** 2022-07-15

**Authors:** Shirley Sze, Daniel Pan, Alastair J. Moss, Cheng Ken Ong, Manish Pareek, Iain B. Squire, Andrew L. Clark

**Affiliations:** ^1^Department of Cardiovascular Science, National Institute for Health Research Leicester Biomedical Research Centre, University of Leicester, Leicester, United Kingdom; ^2^Department of Respiratory Sciences, University of Leicester, Leicester, United Kingdom; ^3^Department of Infectious Diseases and HIV Medicine, University Hospitals of Leicester NHS Trust, Leicester, United Kingdom; ^4^Department of Cardiology, Russell’s Hall Hospital, Dudley Group NHS Trust, Dudley, United Kingdom; ^5^Department of Cardiology, Castle Hill Hospital, Hull University Hospitals NHS Trust, Hull, United Kingdom

**Keywords:** long COVID, ergoreflex, pathophyisology, breathlessness, fatigue, heart failure

## Abstract

Long COVID refers to a multitude of symptoms that persist long after SARS-CoV-2 infection. Fatigue and breathlessness are the most common symptoms of long COVID across a range of studies. They are also cardinal symptoms of chronic heart failure (CHF). In this review, we propose that fatigue and breathlessness in patients with long COVID may be explained by skeletal muscle abnormalities, in a manner similar to patients with CHF. The ergoreflex is a cardiorespiratory reflex activated by exercise, which couples ventilation and cardiovascular function to exercise intensity. At least part of the symptomatology of CHF is related to abnormal skeletal muscle and an enhanced ergoreflex, resulting in heightened sympathetic, vasoconstrictor and ventilator drives. Similarly, SARS-CoV-2 infection results in a hyperinflammatory and hypercatabolic state. This leads to reduction in skeletal muscle mass and altered function. We postulate that the ergoreflex is chronically overstimulated, resulting in fatigue and breathlessness. Exercise training preserves muscle mass and function as well as reduces ergoreflex activation; therefore may have a role in improving symptoms associated with long COVID. Should the ergoreflex be proven to be an important pathophysiological mechanism of long COVID, tailored exercise interventions should be trialed with the aim of improving both symptoms and perhaps outcomes in patients with long COVID.

## Introduction

COVID-19 is a multisystem disease, affecting lungs, digestive tract, kidneys, heart, endocrine system and brain (1, 2). Long COVID refers to a multitude of symptoms that persist long after initial infection. Currently, National Institute for Health and Care Excellence (NICE) defines long COVID as ongoing signs and symptoms beyond 4 weeks after acute COVID-19; whilst the World Health Organisation (WHO) defines it as persistent symptoms 3 months following acute infection that last for at least 2 months and cannot be explained by an alternative diagnosis (3, 4).

Long COVID is a significant challenge for patients, physicians and society. Multiple mechanisms have been proposed to explain the pathophysiology of long COVID. These include viral persistence in certain tissues, immune dysregulation, SARS-CoV2 interactions with host microbiome/virome communities, chronic inflammation, prolonged prothrombotic state, and dysfunctional brainstem/vagus nerve signaling (1, 2). However, the exact etiology of long COVID remains unclear, and the patient profile and symptom patterns are variable and difficult to define with precision. Despite this, two symptoms—fatigue and breathlessness—are consistently the most common symptoms described in observation studies (5). The European Society for Clinical Microbiology and Infectious Diseases reports the prevalence of fatigue and breathlessness in long COVID to be 31–58% and 24–40%, respectively (6). Here, we propose that fatigue and breathlessness in patients with long COVID may be explained by skeletal muscle abnormalities, in a manner similar to patients with chronic heart failure (CHF).

## Breathlessness and fatigue in chronic heart failure

To understand how skeletal muscle abnormalities may contribute to the development of breathlessness and fatigue in patients with long COVID, we will begin by examining how these symptoms arise in the context of CHF.

Fatigue and breathlessness are the dominant symptoms of patients with CHF. Traditionally, the pathophysiology underlying the symptoms was thought to be a consequence of inadequate cardiac pump function. Low cardiac output leads to abnormal muscle perfusion and signals are then transmitted to the brain which are interpreted as fatigue. In order to maintain cardiac output, the failing heart adapts by increasing left ventricular filling pressure. This leads to a rise in pulmonary venous pressure with pulmonary congestion often presenting as breathlessness. If this chain of events were to explain completely the symptoms of fatigue and breathlessness, then the severity of symptoms should be directly related to the severity of left ventricular systolic dysfunction. However, there is no relation between any measure of central hemodynamic function and exercise performance. In the last 30 years, a large body of research has demonstrated the importance of pathophysiological changes in the periphery as being responsible for the generation of fatigue and breathlessness.

## The ergoreflex

The ergoreflex is a cardiorespiratory reflex activated by exercise which couples ventilation and cardiovascular function to exercise intensity. The existence of a reflex triggered by muscle activity was proposed in 1937 by Alam and Smirk (7). Healthy volunteers performed dynamic exercise while blood vessels draining the exercising limbs were occluded by a sphygmomanometer cuff. The exercise lasted for 4 min and circulatory occlusion was maintained for another 11 min. In the recovery period whilst circulatory occlusion was present, the rise in blood pressure reached during exercise was maintained and further increased after another 3–4 min. There was also a sustained increase in heart rate during circulatory occlusion. Blood pressure and heart rate dropped after removal of circulatory occlusion. It has been posited that a reflex triggered by accumulation of metabolites in exercising muscles is able to influence hemodynamic function; the “metaboreflex.” The metaboreflex causes blood pressure to rise to ensure adequate perfusion of the exercising muscle. Animal models show that mechanical stimulation of muscles and tendons also leads to increase heart rate and blood pressure; this is known as the “mechanoreflex” (8).

**FIGURE 1:**
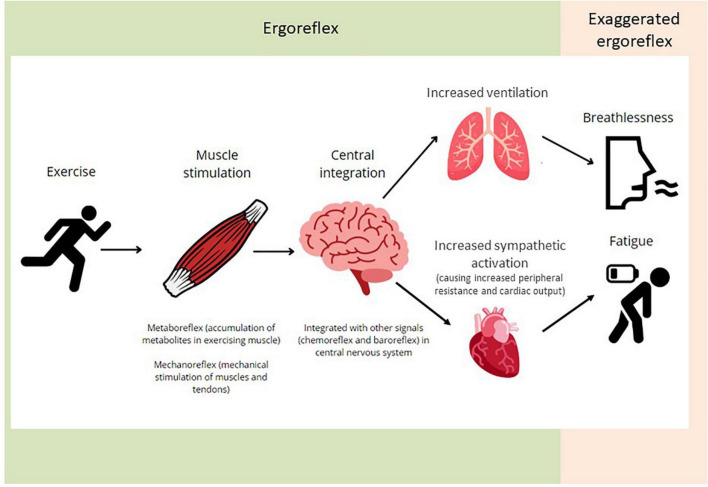
An illustration of the ergoreflex. Skeletal muscle exercise causes stimulation of the metabo- and mechano-receptors. The signals are integrated centrally and contribute to the ventilatory and cardiovascular responses to exercise. Exaggerated ergoreflex (e.g., heart failure) leads to excessive response relative to work performed, leading to the sensation of breathlessness and fatigue.

The combination of the “mechanoreflex” and “metaboreflex” forms the ergoreflex. The mechanoreflex is activated at the beginning of exercise by mechanical stretching of the muscles and tendons. Afferent stimuli are transmitted rapidly *via* thinly myelinated group III fibers in the muscle interstitial space. The metaboreflex is activated by accumulation of metabolites in the exercising muscle, including lactate, hydrogen, and potassium ions, prostaglandins and bradykinin. These are sensed by receptors in the muscle interstitial space, e.g., acid-sensing ion channels, cannabinoid receptors and μ-opioid receptors. Afferent stimuli are transmitted *via* small non-myelinated group IV fibers with a period of latency. Signals from both components integrate with other peripheral and central signals (such as the chemoreflex and baroreflex) in the central nervous system. The efferent limb of the reflex results in increased ventilation and sympathetic activation, in turn causing peripheral resistance and cardiac output to rise, thereby maintaining systemic blood pressure (Figure 1) (8).

## The ergoreflex and heart failure

At least part of the symptomatology of CHF is related to abnormal muscle and an enhanced ergoreflex. Skeletal muscle loss and dysfunction is common, resulting in sarcopenia and cachexia, both of which are associated with poor clinical outcomes (9). Histologically, patients with CHF have a shift in muscle fiber distribution from aerobic type I fibers to anaerobic type II fibers. Mitochondrial structure is also abnormal, with a reduction in the volume of cristae and fall in the enzymes of the Krebs cycle (10). Cardiac dysfunction leads to abnormal muscle physiology *via* several mechanisms including the release of proinflammatory cytokines, mitochondrial dysfunction, physical inactivity as a result of lower exercise tolerance, intestinal congestion and malnutrition. There is resistance to pro-anabolic hormones such as insulin. Skeletal myopathy increases ergoreflex sensitivity, leading to exertional breathlessness due to a greater ventilatory response to a given amount of exercise. Chronic sympathetic activation also results in peripheral vasoconstriction and increased cardiac afterload, leading to a vicious cycle of progressive muscle and cardiac dysfunction (10).

**FIGURE 2:**
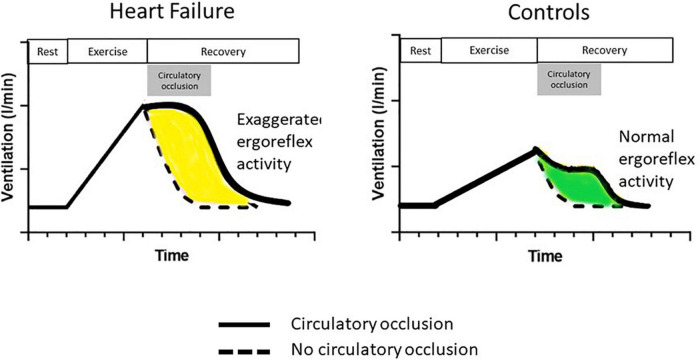
The ergoreflex in chronic heart failure. Following handgrip dynamometer exercise to exhaustion, a cuff is either inflated around the exercising arm at peak exercise (solid line) or not (dotted line). The cuff is deflated after 3 min (end of circulatory occlusion in gray box). Compared to controls, patients with heart failure have an exaggerated ergoreflex response, resulting in greater ventilatory response to exercise. Their ventilatory response is maintained at the same level as maximum exertion throughout circulatory occlusion. Adapted from Piepoli et al. (11).

Piepoli et al. examined the ergoreflex in patients with CHF. Subjects performed handgrip exercise using their non-dominant arm by performing two 5-min handgrip manoeuvers at approximately 50% of pre-determined maximal contraction, in random order, separated by 30 min rest: one bout with circulatory occlusion during the last 10 s of exercise and the first 3 min of recovery (“clamp session”) and one bout with no occlusion (Figure 2). Ergoreceptor sensitivity was quantified as the percentage of the ventilatory and hemodynamic response to exercise maintained by circulatory occlusion during the third minute of recovery, compared with the third minute of recovery without occlusion (11).

Patients with CHF had a much greater ergoreflex than controls with heightened sympathetic, vasoconstrictor and ventilatory drives (Figure 2). Interestingly, these abnormalities are potentially reversible. After 6 weeks of forearm training, there was a marked reduction in ergoreflex activity. These findings perhaps underlie some of the beneficial effects of exercise training in CHF: an improvement in muscle structure and function by exercise training can reduce ergoreflex sensitivity, thereby leading to a reduction in symptoms (11).

## Skeletal muscle changes and ergoreflex activation in long COVID

Acute SARS-CoV-2 infection results in a hypercatabolic state (12, 13). Firstly, insulin resistance is common in patients with COVID-19 during acute infection (14). As a consequence of the RECOVERY trial, universal treatment with corticosteroids for patients hospitalized with COVID-19 requiring oxygen has decreased mortality from acute infection but also predisposed patients to the development of insulin resistance (15, 16). Insulin resistance and the development of diabetes are common in patients with long COVID, both of which are characterized by high circulating concentrations of insulin and normal fasting glucose (17). Secondly, a cytokine storm during COVID-19 infection also results in excessive cortisol secretion in the first 2 weeks of acute illness, causing sympathetic overactivation (18, 19). Thirdly, SARS-CoV-2 uses the angiotensin-converting enzyme-2 (ACE-2) to facilitate cell entry, which might cause stimulation of the renin-angiotensin-aldosterone system (RAAS), predisposing to chronic inflammation and hypercatabolism (20). The mechanism by which age, obesity, hypertension and diabetes are risk factors in COVID infection might relate to RAAS activation (21).

A chronic catabolic state in infected individuals may lead to long-term reduction in skeletal muscle mass and altered function, predisposing to the development of long COVID. In a study of 213 patients with COVID-19, during the acute phase of infection, 29% of patients lost over 5% of their body weight (median percentage weight loss = 8.1%, 95% confidence interval = 6.1–10.9%). The weight loss observed may be due to a combination of acute inflammatory state and disuse atrophy (22). Sarcopenia may develop within a matter of days or insidiously over months and years. Patients admitted to an intensive care unit due to COVID-19 have a median reduction of 30% in their rectus femoris cross sectional area and 19% in the thickness of the anterior compartment of the quadricep muscle between the first and tenth day of their intensive care admission (23). Sarcopenia may contribute to fatigue, the extent of which depends on disease severity. In a study of 807 people with long COVID 1 year following acute infection, 7 of 10 most common persistent symptoms could be explained by sarcopenia (fatigue, aching muscles, physically slowing down, breathlessness, joint swelling or pain, general pain and limb weakness). Proteomic analysis shows that increased inflammatory mediators of tissue damage and repair are associated with the most severe symptoms (24).

**FIGURE 3:**
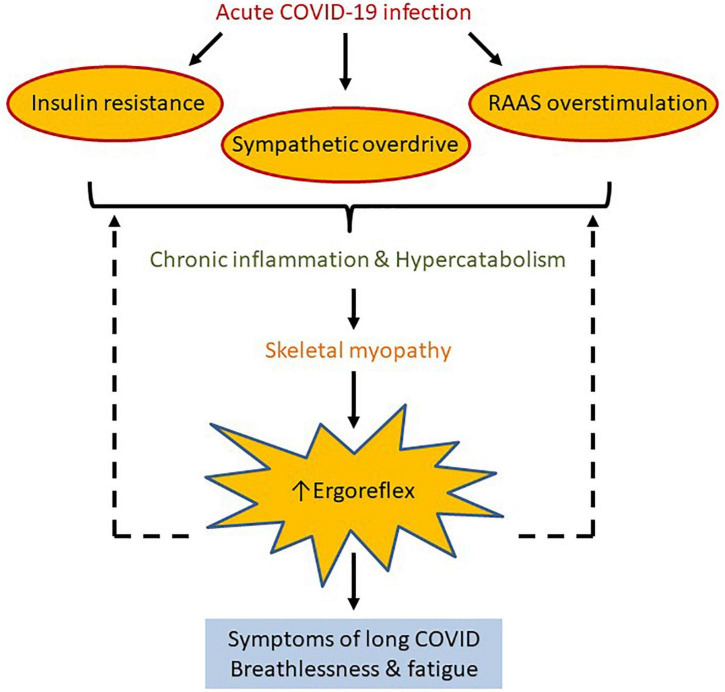
We propose ergoreflex overstimulation as a possible pathophysiological mechanism of long COVID. Acute COVID-19 infection predisposes to insulin resistance and sympathetic and renin-angiotensin-aldosterone system overstimulation, which may lead to chronic inflammation and hypercatabolism. This in turn can cause reduction in skeletal mass and function, which increases ergoreflex sensitivity, and perhaps explains the symptoms of long COVID such as breathlessness and fatigue. Solid and dotted arrows represent known and hypothetical relations, respectively.

A unifying hypothesis to explain breathlessness and fatigue in patients with long COVID is that skeletal muscle becomes abnormal in some patients following acute infection, secondary to hyperinflammatory and hypercatabolic response (Figure 3). Excessive ergorflex activation leads to the sensations of fatigue and breathlessness.

## Future research

To prove this hypothesis, future work should aim to characterize muscle changes in patients with prior COVID-19 infection, comparing those with and without long COVID. The prevalence and severity of sarcopenia in people with long COVID should be investigated and quantified in more detail. Widespread availability of dual energy X-ray absorptiometry scanning is a straightforward way to assess the problem of loss of muscle bulk. The ergoreflex itself can be directly examined using the protocol developed and standardized by Piepoli et al. (11).

Although COVID vaccination reduces the risk of developing COVID-19 and associated disease severity, its relation with long COVID is unknown (25). Comparing the muscle characteristics and ergoreflex response in vaccinated vs. non-vaccinated individuals may help understand whether vaccines could lower the risk of developing long COVID and severity of symptoms.

Importantly, the ergoreflex hypothesis supports the use of exercise training to improve symptoms in patients with long COVID. By preserving the bulk and functioning of large muscle groups, as well as reducing ergoreflex activation, we postulate that exercise training may improve symptoms associated with long COVID. Indeed, despite very severe left ventricular dysfunction, some patients with CHF have normal exercise responses (due to preserved muscle bulk) and are asymptomatic. Should the ergoreflex be proven to be an important pathophysiological mechanism of long COVID, tailored exercise interventions should be trialed with the aim of improving both symptoms and perhaps prognosis in patients with long COVID.

## Author contributions

SS and DP conceived of the presented idea and drafted the manuscript. AM, CO, MP, IS, and AC provided feedback and commented on the manuscript. All authors contributed to the article and approved the submitted version.

## Conflict of Interest

The authors declare that the research was conducted in the absence of any commercial or financial relationships that could be construed as a potential conflict of interest.

## Publisher’s Note

All claims expressed in this article are solely those of the authors and do not necessarily represent those of their affiliated organizations, or those of the publisher, the editors and the reviewers. Any product that may be evaluated in this article, or claim that may be made by its manufacturer, is not guaranteed or endorsed by the publisher.
